# Formulation and Characterization of Carbopol-Based Porphyrin Gels for Targeted Dermato-Oncological Therapy: Physicochemical and Pharmacotechnical Insights

**DOI:** 10.3390/ijms26083641

**Published:** 2025-04-11

**Authors:** Emma Adriana Ozon, Mihai Anastasescu, Adina Magdalena Musuc, Andreea Mihaela Burloiu, Radu Petre Socoteanu, Irina Atkinson, Raul-Augustin Mitran, Daniela C. Culita, Dumitru Lupuliasa, Dragos Paul Mihai, Cerasela Elena Gird, Rica Boscencu

**Affiliations:** 1 Faculty of Pharmacy, “Carol Davila” University of Medicine and Pharmacy, 6 Traian Vuia St., 020956 Bucharest, Romania; emma.budura@umfcd.ro (E.A.O.); dumitru.lupuliasa@umfcd.ro (D.L.); dragos_mihai@umfcd.ro (D.P.M.); cerasela.gird@umfcd.ro (C.E.G.); rica.boscencu@umfcd.ro (R.B.); 2 Institute of Physical Chemistry—Ilie Murgulescu, Romanian Academy, 202 Spl. Independentei, 060021 Bucharest, Romania; manastasescu@icf.ro (M.A.); psradu@yahoo.com (R.P.S.); iatkinson@icf.ro (I.A.); rmitran@icf.ro (R.-A.M.);

**Keywords:** hydrogel, biomedical applications, unsymmetrical porphyrin, Carbopol, photodynamic therapy

## Abstract

Malignant skin conditions are classified as the most common forms of cancer, with an evolution of one million new cases reported every year. Research efforts in the medical field are focused on developing innovative strategies for the dissemination of measures for preventing cancer and providing new antitumor compounds. The present research examines the development and evaluation of 1% Carbopol-based hydrogels incorporating two porphyrin derivatives—5,10,15,20-tetrakis-(4-acetoxy-3-methoxyphenyl) porphyrin (P2.1) and 5-(4-hydroxy-3-methoxyphenyl)-10,15,20-tris-(4-acetoxy-3-methoxyphenyl) porphyrin (P2.2)—to create formulations suitable for topical photodynamic therapy (PDT) applications. The physicochemical properties of the obtained hydrogels were carefully evaluated, revealing the successful integration of the porphyrins into the 1% Carbopol hydrogel matrix. Rheological analysis demonstrated pseudoplastic behavior, with an increase in viscosity properties for P2.1 and P2.2, suggesting interactions with the Carbopol polymer structure. UV-visible and fluorescence spectroscopy confirmed the maintenance of the porphyrins’ photodynamic properties, essential for therapeutic efficacy. Pharmacotechnical studies highlighted the hydrogels’ suitability for topical applications. The formulations maintained an optimal pH range, ensuring skin compatibility and minimizing the potential for skin irritation. Their mechanical properties, including elasticity and rigidity, provided stability during handling and application. The high swelling capacity indicated effective moisture retention, enhancing skin hydration and drug release potential. Furthermore, the hydrogels demonstrated excellent spreadability, enabling uniform application and coverage, crucial for efficient light activation of the photosensitizers. The combination of robust physicochemical and pharmacotechnical properties highlights the potential of these porphyrin-loaded 1% Carbopol hydrogels as promising carriers for topical PDT. These results permit further biological and therapeutic investigations to optimize the formulation for clinical use, advancing the development of effective localized photodynamic therapies.

## 1. Introduction

Malignant cutaneous disorders are included by world statistics among the most common types of cancer in humans globally, with over one million new cases reported and diagnosed annually [1,2,3]. One of the therapeutic strategies in the management of premalignant and malignant skin conditions is the topical treatment applied in the early stage of the disease. This approach offers the advantage and the possibility of treating larger areas of precancerous lesions [4]. However, the therapeutic effectiveness of topical delivery systems is often hindered or diminished by poor penetration of the active compound into the tumor microenvironment and the need for prolonged treatment, which can lead to notable potential for local side effects.

Photodynamic therapy (PDT) has developed as a modern approach for treating localized diseases, especially skin cancers, utilizing photosensitizers that, upon light activation, generate reactive oxygen species (ROS) to selectively destroy the targeted tissues [5]. This technique employs photosensitizers structurally close to protoporphyrin IX, a metabolite of aminolevulinic acid (ALA), and offers advantages such as field PDT application and a low risk of systemic phototoxicity. Despite their promising attributes—including selectivity for cell tumors, low cytotoxicity in the absence of light, and excellent spectral properties—protoporphyrin-IX-based photosensitizers are limited by their low accumulation rates in the skin’s deep tissue. The skin’s barrier function, which is designed to prevent the absorption of exogenous substances, contributes to the challenge of achieving effective photosensitizer delivery to deeper tissues. Additionally, the hydrophilic nature of protoporphyrin IX further complicates its permeation across the lipophilic stratum corneum. As a result, the photosensitizer often accumulates primarily in the epidermal and superficial dermal layers, limiting its therapeutic efficacy, especially in treating deep skin cancers or lesions. The optimization of photosensitizer molecules involves chemo- and regioselective modifications, wherein functional groups with varying polarities are strategically attached to the tetrapyrrole ring. These modifications enhance solubility in biological environments, reduce the tendency for molecular aggregation, and improve photodynamic efficiency for PDT. These advancements align with findings from the literature and are supported by conclusions from our earlier studies [6,7,8].

To address these challenges, the incorporation of porphyrin photosensitizers into suitable delivery systems is essential. Versatile structured systems, including polymer-based hydrogels, are increasingly utilized among the most used matrices to enhance the delivery of photosensitizers for diagnosis and tumor therapy [9]. Natural polymers, particularly in the class of cellulose derivatives, have shown significant potential as carriers. These macromolecular compounds, with their structural profile compatible with porphyrinic-type tetrapyrrole compounds, provide improved transport of photosensitizers to tumor cells by reducing the molecular aggregation potential of the active substance [10,11]. Recent advancements in drug delivery systems, such as the development of polymeric gels and hydrogels, have shown potential in enhancing the penetration and retention of protoporphyrin IX in the skin. These systems, when properly optimized, can overcome the skin barrier by improving the solubility, stability, and controlled release of the photosensitizer, potentially leading to better tissue distribution and improved therapeutic results in PDT [12,13,14,15].

Natural polymer-based hydrogel systems offer advantages such as enhanced bioavailability, biocompatibility, biodegradability, and the ability to maintain a moist wound-healing environment favorable to wound healing [16,17,18,19]. Incorporating porphyrin photosensitizers into these hydrogels allows the development of controlled-release systems, improving, at the same time, the stability and bioavailability of photosensitizers while enabling localized delivery for topical PDT.

Porphyrins are macrocyclic compounds with a tetrapyrrole structure that closely resembles the heme group of hemoglobin. Their conjugated π-electron system allows them to absorb light strongly in the therapeutic window (600–800 nm), enabling deep tissue penetration. Additionally, porphyrins exhibit fluorescence, making them dual-functional agents for both therapeutic (PDT) and diagnostic (imaging) applications, often referred to as theranostics. This dual capability enhances their potential in oncology, where real-time monitoring of drug localization is crucial [20,21,22].

This study focuses on formulating two selected porphyrin photosensitizers, i.e., 5,10,15,20-tetrakis-(4-acetoxy-3-methoxyphenyl) porphyrin (P2.1) and 5-(4-hydroxy-3-methoxyphenyl)-10,15,20-tris-(4-acetoxy-3-methoxyphenyl) porphyrin (P2.2), for oncological dermatological applications by incorporating them into hydrogel matrices of Carbopol 940 (noted as C-P2.1 and C-P2.2, respectively). P2.1 and P2.2 have proven strong photochemical and biological activity, establishing their potential as promising photosensitizers for photodynamic therapy [23,24]. Particularly, P2.2 demonstrates excellent potential as a theranostic agent for photodynamic therapy in solid tumors, exhibiting good solubility in biologically compatible media, preferential accumulation in tumor cells over blood cells, and strong fluorescence suitable for imaging applications. Furthermore, P2.2 generates singlet oxygen at yields effective for PDT while exhibiting minimal in vitro cytotoxicity toward tumor-specific cells, such as colon carcinoma cells and tumorigenic fibroblasts, as well as peripheral blood mononuclear cells (PBMC). These characteristics highlight the suitability of both porphyrin derivatives for further investigation in PDT applications [24]. As a novelty, this research aims to provide a new system able to enhance the therapeutic potential of PDT by overcoming the limitations associated with photosensitizer delivery and stability.

Carbopol—a synthetic high-molecular-weight polymer of acrylic acid cross-linked with polyalkenyl ethers—is widely used as a gelling agent in pharmaceutical formulations due to its high viscosity and stability [25]. Carbopol gels are characterized by their high viscosity, excellent spreadability, bioadhesive properties, and ability to maintain drug stability within the formulation. Additionally, Carbopol’s compatibility with various active pharmaceutical ingredients and its ability to form a stable gel at low concentrations make it an ideal matrix for topical drug delivery. In the context of photodynamic therapy, incorporating porphyrin derivatives into Carbopol gels can enhance their therapeutic efficacy. This study presents the complete physicochemical characterization using Fourier-transform infrared spectroscopy (FTIR), X-ray diffraction (XRD), UV-vis, atomic force microscopy (AFM), and thermal analyses (TGA) of 1% Carbopol gels incorporating the porphyrin derivatives P2.1 and P2.2, comparing the findings with existing literature. Also, the pharmacotechnical and rheological properties of the new systems based on the developed porphyrin-doped hydrogels are evaluated for their suitability for biomedical applications and, in particular, as promising carriers for topical photodynamic therapy.

## 2. Results and Discussion

### 2.1. Physicochemical Characteristics

#### 2.1.1. Appearance of the Hydrogels

The resulting hydrogels were transparent, sticky, free of visible air bubbles, and slightly pink in color. The density of the 1% Carbopol gel was 1.21 g/mL and the densities of C-P2.1 and C-P2.2 were 1.03 g/mL and 1.07 g/mL, respectively.

#### 2.1.2. FTIR Analysis

**Figure 1:**
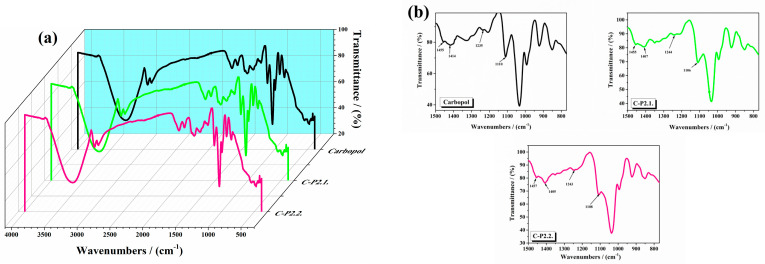
The FTIR spectra of the studied hydrogels (**a**) between 4000 and 500 cm^−1^ and (**b**) between 1500 and 770 cm^−1^.

The FTIR spectra of the analyzed hydrogels are displayed in Figure 1 ((a) between 4000 and 500 cm^−1^ and (b) between 1500 and 770 cm^−1^—black line: 1% Carbopol gel, green line: C-P2.1, and pink line: C-P2.2). The FTIR spectrum of the 1% Carbopol gel shows peaks at 3270 cm^−1^ due to O-H stretching, at 1710 cm^−1^ (C=O stretching), indicative of the carboxyl groups present in the polyacrylic acid backbone of Carbopol, at 1417 cm^−1^ (O-H bending), corresponding to the bending vibrations of hydroxyl groups, which are part of the cross-linked network, and between 1050 and 880 cm^−1^ (C-C-O symmetric and antisymmetric stretching), associated with the stretching vibrations of the C-O bond in the carboxylate group. C-P2.1 and C-P2.2 show slight shifts in the C=O stretching region and changes in O-H bending peaks.

The literature reports that pure Carbopol shows O-H bending around 1455 cm^−1^, and peaks around 1414, 1235, and 1110 cm^−1^, which are also presented in the spectrum of the 1% Carbopol gel [26,27]. Shifts in these peaks and their modified intensity upon drug incorporation indicate potential interactions, such as hydrogen bonding and electrostatic interactions, between the carboxyl groups of Carbopol and the acetoxy groups of P2.1. The observed shifts in the absorption bands suggest that the porphyrins interact with the Carbopol matrix through various mechanisms, likely due to porphyrin–polymer interactions. These include hydrogen bonding, where the carboxyl and hydroxyl groups of Carbopol form hydrogen bonds with the porphyrin molecules, stabilizing the incorporation of the photosensitizers into the polymer network. However, these changes do not necessarily translate into an increase in viscosity. Our results are consistent with these observations, confirming the successful incorporation and interaction of porphyrins with Carbopol. The literature documents that a shift in the C=O stretching region in polymer–drug systems often indicates interactions (such as hydrogen bonding or ionic interactions) between the drug and the polymer matrix [28]. The observed shifts to 1648 cm^−1^ (P2.1) and 1647 cm^−1^ (P2.2) in our study align with these findings, confirming the presence of such interactions. The broadening of the O-H bending peak at 1450 cm^−1^ indicates enhanced hydrogen bonding within the gel matrix. Literature on polymeric gels incorporating hydrophilic drugs also reports similar broadening, attributing it to increased hydrogen bond formation due to drug–polymer interactions [29]. The FTIR spectra of the 1% Carbopol gels incorporating the porphyrin derivatives P2.1 and P2.2 do not reveal significant structural changes. Shifts in the C=O stretching region and the broadening of the O-H bending peak suggest successful incorporation and interaction of the porphyrins within the Carbopol gel [23,24].

#### 2.1.3. XRD Analysis

**Figure 2:**
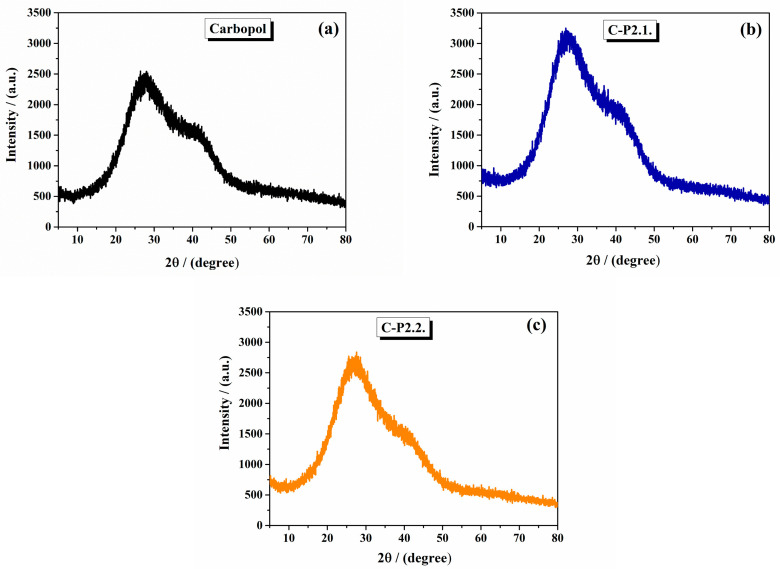
The X-ray diffractograms of (**a**) 1% Carbopol gel, (**b**) C-P2.1, and (**c**) C-P2.2.

The XRD diffraction spectra of the 1% Carbopol gel, C-P2.1, and C-P2.2 are shown in Figure 2. The 1% Carbopol gel has an amorphous pattern with a broad peak around 20°, indicating the amorphous nature of Carbopol (Figure 2a). This feature reflects the lack of long-range order in the cross-linked polyacrylic acid structure, consistent with literature reports for Carbopol polymers [30]. C-P2.1 (Figure 2b) and C-P2.2 (Figure 2c) show slight changes in their peak patterns at specific 2θ values, but the changes are not significant.

From the literature, Carbopol typically exhibits an amorphous structure with a broad peak in the XRD pattern around 27° and 41° [31].

#### 2.1.4. Thermal Measurements

**Figure 3:**
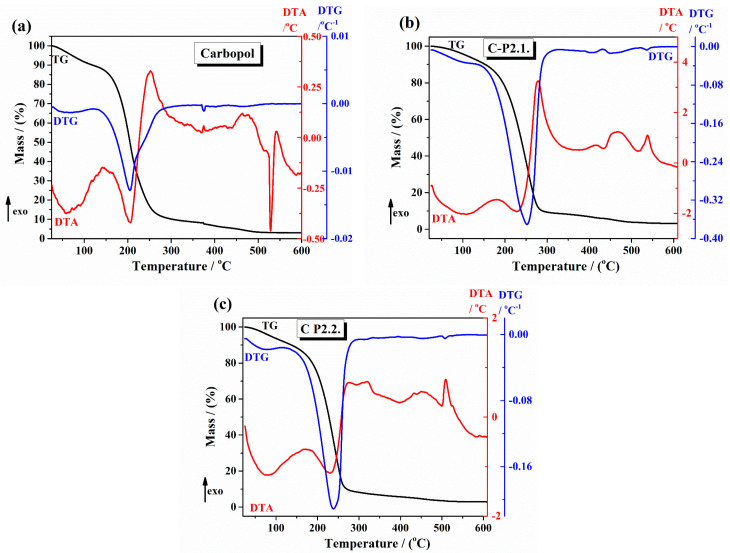
The TG/DTG and DTA curves of (**a**) 1% Carbopol gel, (**b**) C-P2.1, and (**c**) C-P2.2.

The thermal curves of the 1% Carbopol gel and Carbopol integrated with porphyrins gels are presented in Figure 3 ((a) 1% Carbopol gel, (b) C-P2.1, and (c) C-P2.2). The TG curve of the 1% Carbopol gel presents the following thermal steps. First, an initial weight loss below 150 °C due to the loss of adsorbed water, associated with an endothermal effect on the DTA curve. Second, Figure 3a shows, on its thermal curve, a major weight loss starting around 150 °C and corresponding to the thermal degradation of the Carbopol polymer backbone [32]. The thermal curves of C-P2.1 (Figure 3b) present a major two-step weight loss in two stages: the first step starting around 180 °C and the second step around 310 °C, associated with exothermal effects on TGA curves. C-P2.2 (Figure 3c) also shows a two-step weight loss pattern starting at 190 °C. TGA studies in the literature often show a major weight loss for Carbopol starting around 200–230 °C, caused by the combustion of organic components, with variations due to the presence of incorporated drugs or additives [33]. The thermal analysis results are consistent with these reports, indicating that the incorporation of porphyrin derivatives not only interacts with the Carbopol matrix, but also enhances its thermal stability.

The thermal data obtained from TG/DTG-DTA curves regarding the decomposition steps, main temperatures, and mass loss values are shown in Table 1. Taking into account that the developed formulations are used for skin applications (so we take into consideration the body temperatures and also the increasing temperatures by applying local activation by laser irradiation), thermogravimetric analysis (TGA) has proven invaluable in evaluating hydrogels’ thermal stability and decomposition behavior, providing essential insights into their stability under different temperature conditions.

The FTIR, XRD, and thermal analyses of 1% Carbopol gels incorporating the porphyrin derivatives P2.1 and P2.2 provide a comprehensive understanding of their physicochemical properties. These analyses provide insights into the molecular interactions, structural modifications, and thermal behaviors of the gel systems, which are crucial for the development of effective photodynamic therapy formulations for treating malign and non-malignant skin diseases. The physicochemical properties of the 1% Carbopol gels with the porphyrin derivatives P2.1 and P2.2 make them promising candidates for topical PDT:

(i) The modified thermal properties suggest that the gels are more stable and could provide controlled release of the porphyrin derivatives;
(ii) The observed interactions and structural modifications ensure that the porphyrin derivatives are well-incorporated and can be effectively delivered to the target sites;
(iii) The strong interactions between the polymers and the porphyrin derivatives support their potential efficacy in treating various diseases through PDT.

#### 2.1.5. AFM Analysis

**Figure 4:**
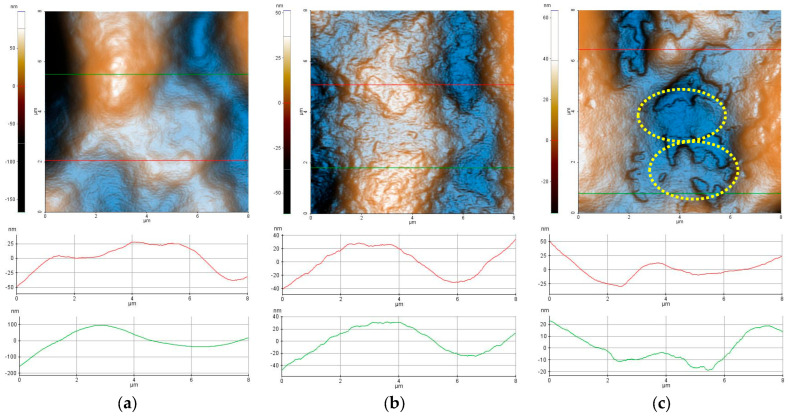
Enhanced-color 2D AFM topographic images of the 1% Carbopol (**a**), C-P2.1 (**b**), and C-P2.2 (**c**) samples recorded at the scale of 8 µm × 8 µm. Below each AFM image, two characteristic surface profiles (red and green scan lines) are exemplified.

Figure 4 presents the two-dimensional (2D) AFM topographic images, in enhanced color mode, of the hydrogels, based on Carbopol (1% Carbopol, C-P2.1, and C-P2.2), recorded at a scale of 8 µm × 8 µm, accompanied by characteristic profile lines (scanning lines collected from the AFM at the positions indicated by the horizontal red/green lines in the AFM images). From Figure 4a it can be seen that the hydrogel based on Carbopol (1% Carbopol) presents a continuous and compact surface with a corrugation of the order of tens to hundreds of nm (see the two profile lines in Figure 4a). For example, the red line (taken from the lower half of the AFM image—Figure 4a) has roughness on the vertical level difference along the z-axis of about 75 nm (from −50 nm to +25 nm), while the green line has a corrugation of about 300 nm (from −200 nm to +100 nm). The 1% Carbopol sample is characterized by an RMS roughness (Rq) of 38.8 nm and a peak-to-valley parameter (Rpv) of about 265.3 nm—the highest values in the series of Carbopol-based hydrogels. The incorporation of porphyrin P2.1 into the 1% Carbopol hydrogel (Figure 4b) leads to the formation of a surface with a more textured appearance, due to the presence of “irregular particle-like asperities” (protrusions on the nanometric scale).

The profile line shown in Figure 4b has a vertical corrugation of approximately 80 nm (from −40 nm to +40 nm). The C-P2.1 sample is characterized by an RMS roughness (Rq) of 18.8 nm and a peak-to-valley parameter (Rpv) of about 112.7 nm. Finally, the incorporation of porphyrin P2.2 in the 1% Carbopol hydrogel (Figure 4c) changes the morphology of C-P2.2 in such a way that, in certain areas, the sample resembles the 1% Carbopol hydrogel, but additional shallow cavities appear, with edges exhibiting a disordered aspect (see the areas marked by dotted yellow lines). The C-P2.2 sample is characterized by an RMS roughness (Rq) of 19.9 nm and a peak-to-valley parameter (Rpv) of about 99.0 nm.

**Figure 5:**
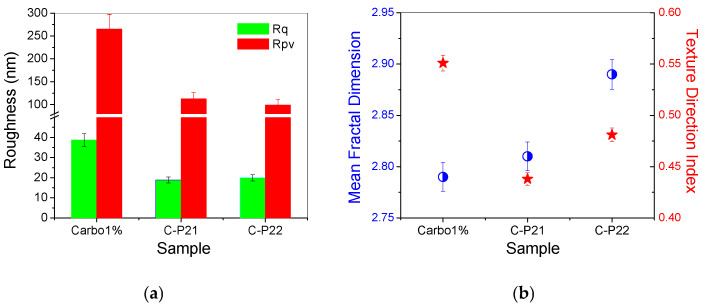
(**a**) Corrugation parameters: RMS roughness (Rq) and peak-to-valley parameter (Rpv); (**b**) mean fractal dimension (MFD) and textural direction index (Stdi) for the hydrogels based on Carbopol (1% Carbopol, C-P2.1, and C-P2.2) at the scale of 8 µm × 8 μm.

The corrugation parameters of the Carbopol-based hydrogel samples (evaluated from the AFM images scanned over 8 × 8 µm^2^) are presented in the form of histograms in Figure 5a, showing the decrease in the peak-to-valley parameter in the order of 1% Carbopol > C-P2.1 > C-P2.2. In a previous study, we found that the P2.1 porphyrin exhibits a high tendency of aggregation [23]. Note that Rpv is the most suggestive parameter for practical applications, as it provides the dimension of the vertical corrugation of the samples. Figure 5b shows the values of the mean fractal dimension (MFD) and the textural direction index (Stdi). It is noted that the C-P2.1 and C-P2.2 samples have a slight tendency for anisotropy (the physical properties may display a slightly different behavior depending on the direction), as suggested by the Stdi values, which are lower than 0.5 (with the 1% Carbopol sample having an Stdi index > 0.5).

Concerning the average fractal dimension (Figure 5b—blue circles), there is a tendency to increase the MFD values in the sequence 1% Carbopol < C-P2.1 < C-P2.2. This fact is somewhat unexpected, given that, usually, the MFD values follow the roughness trend. In this series, the roughness decreases (Figure 5a), but the values of the fractal dimension increase, a phenomenon that can only be attributed to the increase in the complexity of the topography of the surfaces that incorporate porphyrins. Fractals can be described as complex objects with fine structures at arbitrarily small scales, exhibiting some degree of approximate or statistical self-similarity [34]. A larger fractal dimension implies greater complexity of the objects studied [35]. Therefore, analyzing a topographic micrograph (AFM images in the present case) by self-similarity requires the quantification of its complexity through the fractal dimension, which is a direct measure of the complexity of the figure, so that the higher the degree of complexity, the larger the fractal dimension [36].

**Figure 6:**
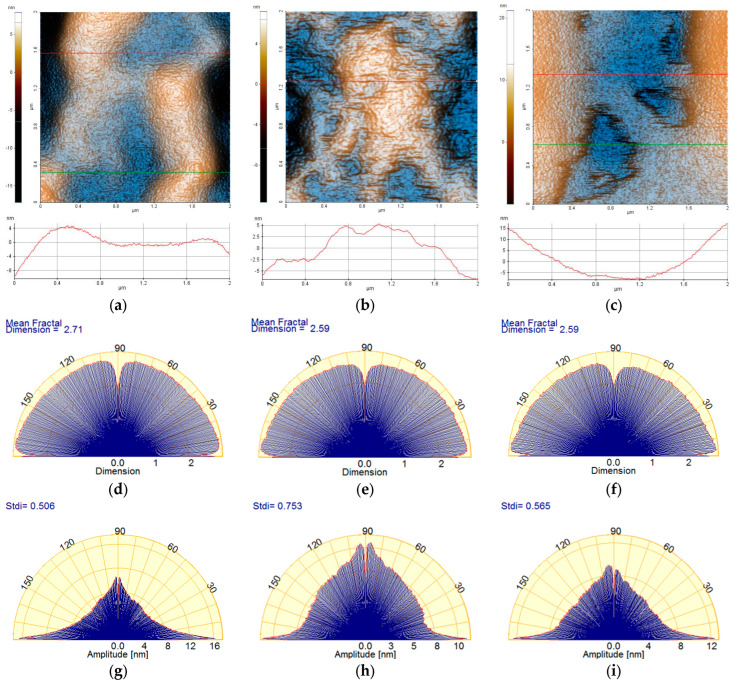
Enhanced-color 2D AFM topographic images of the 1% Carbopol (**a**), C-P2.1 (**b**), and C-P2.2 (**c**) samples at the scale of 2 µm × 2 µm; below each AFM image, the characteristic profiles (scan lines) are exemplified. Angular Fourier spectra of the mean fractal dimension (MFD) (**d**–**f**) and textural direction index (Stdi) (**g**–**i**) were plotted for the AFM images of the 1% Carbopol, C-P2.1, and C-P2.2 samples at the scale of 2 µm × 2 µm.

Figure 6 highlights the morphological details of the Carbopol-based hydrogels by recording the AFM images at a lower scale, namely 2 µm × 2 µm. In the case of the 1% Carbopol sample (Figure 6a), the presence of a nanometric texture (similar to an “orange peel” type texture) is noticeable. The constituent bumps (an alternance of “particles” and “pores”) are of the order of a few nm in the horizontal plane, and no more than 1 nm vertically. At the observation scale (2 × 2 µm^2^), the 1% Carbopol sample is characterized by an RMS roughness (Rq) of 3.3 nm and a peak-to-valley parameter (Rpv) of about 25.1 nm (note a decrease by one order of magnitude in the roughness parameters in comparison with those evaluated at the scale of 8 × 8 µm^2^). The sample incorporating porphyrin P2.1 exhibits a “wrinkled” texture, as suggested by both the AFM image in Figure 6b and the corresponding line profile that shows alternating peaks/valleys of the order of a few nm (the z-axis having values between −5 nm and +5 nm). At the observation scale (2 × 2 µm^2^), the C-P2.1 sample is characterized by an RMS roughness (Rq) of 3.2 nm and a peak-to-valley parameter (Rpv) of about 18.8 nm. Finally, the C-P2.2 sample (Figure 6c) preserves its self-similarity characteristics observed at the upper scale: there are patches texturally similar to the 1% Carbopol hydrogel (orange peel) and the presence of superficial cavities (approximately 4 nm deep) with disordered edges. At the observation scale (2 × 2 µm^2^), the C-P2.2 sample is characterized by an RMS roughness (Rq) of 6.4 nm and a peak-to-valley parameter (Rpv) of about 31.2 nm.

**Figure 7:**
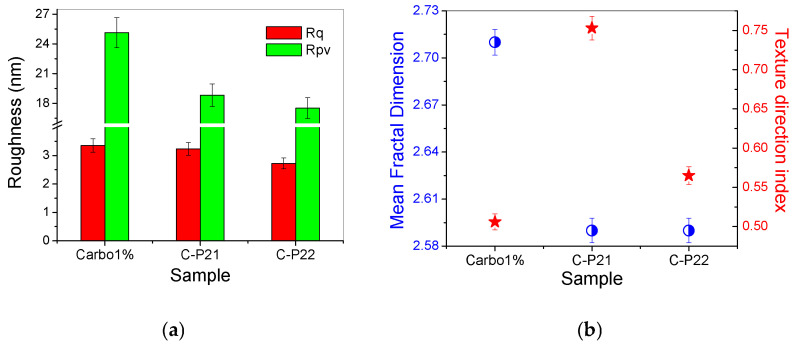
(**a**) Corrugation parameters: RMS roughness (Rq) and peak-to-valley index (Rpv); (**b**) mean fractal dimension (MFD) and textural direction index (Stdi) for the hydrogels based on Carbopol (1% Carbopol, C-P2.1, and C-P2.2) at the scale of 2 µm × 2 µm.

The corrugation parameters of the series of hydrogels based on Carbopol (at the scale of 2 × 2 µm^2^) are summarized in the form of histograms in Figure 7a, displaying the decrease in both the RMS roughness (Rq) and the peak-to-valley parameter (Rpv) in the following order: 1% Carbopol > C-P2.1 > C-P2.2. Figure 7b shows the values of the mean fractal dimension (MFD) and of the textural direction index (Stdi).

The values of MFD and Stdi, assessed from Figure 6a–i, are summarized in Figure 7b. It can be noted from Figure 7b that, at lower scales (2 µm × 2 µm), the Carbopol-based hydrogel samples exceed the value of 0.5 (which is the threshold for the anisotropic–isotropic transition of the Stdi index), but only the C-P2.1 sample shows high isotropic values, while the 1% Carbopol and C-P2.2 samples remain at the lower limit (the physical properties may still exhibit a slightly different behavior depending on the direction).

From the fractal behavior point of view, it can be stated that the samples are self-similar at both scales of observation, but, in this case (2 × 2 μm^2^), the MFD values follow the trend of the surface corrugation, so that the MFD values for the C-P2.1 and C-P2.2 samples are lower in comparison to the MFD values of the hydrogel without porphyrins (1% Carbopol). In other words, the topographical complexity of the hydrogels containing porphyrins decreases at a lower scale (exhibiting a linear behavior in relation to the surface roughness).

#### 2.1.6. UV-Vis and Fluorescence Spectroscopy

The results of the spectral analysis highlighted the preservation of the spectral profile of the two porphyrins after incorporation into the gel, with the retention of absorption and emission maxima in the spectral range relevant for applications in PDT for malignant cutaneous skin disorders [37,38].

**Figure 8:**
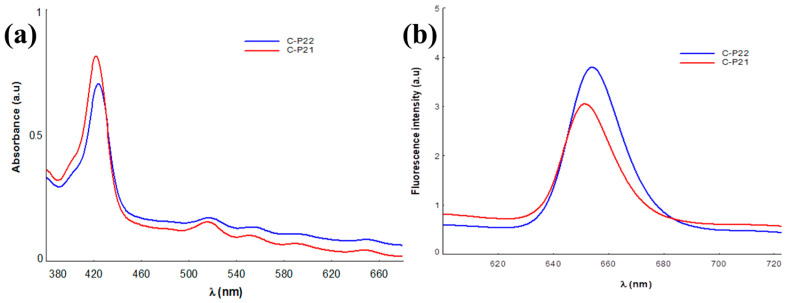
Absorption (**a**) and emission (**b**) spectra of P2.1 and P2.2 in the Carbopol matrix.

The values of the spectral parameters associated with the absorption and fluorescence maxima of the gel–porphyrin samples investigated are presented in Table 2. In Figure 8, the emission spectra of P2.1 and P2.2 in the 1% Carbopol matrix are shown as an example.

The results of the UV-vis spectral analysis confirm the presence of the P2.1 and P2.2 porphyrinic structures in the polymer gels through the absorption maxima values that highlight the Soret band at approximately 427 nm and the four Q bands positioned in the 516–650 nm spectral range [24]. Regarding the emission properties, in the investigated samples, the presence of a strong fluorescent signal, in the spectral range of 650–654 nm, specific to porphyrinic structures can be noted [24,39,40], confirming the maintenance of the spectral characteristics of the porphyrinic photosensitizers through their integration in the Carbopol gel, as our previous studies on unsymmetrical porphyrins demonstrated [24].

It should be noted that, in this formulating framework, the spectral characteristics of the porphyrinic compounds P2.1 and P2.2 do not change significantly compared to those in their solutions in the PEG 200 solvent or dilutions with the phosphate buffer solution (PEG 200/PBS = 1/1000), and will not affect the photosensitizing activity of porphyrin [23].

The results confirm an absorption and emission potential associated with the two Carbopol–porphyrin matrices, optimal for use in PDT at the skin level.

### 2.2. Pharmacotechnical Characteristics

#### 2.2.1. pH Values

The final pH of the 1% carbomer base gel was 6.5, but, as other studies have already shown [41], the pH is significantly increased by the addition of porphyrins to the gel matrix. The pH value of C-P2.1 is 7.3 and that of C-P2.2 is 7.4. Essentially, the final pH of the hydrogels ensures good tolerability, and they do not cause irritation when applied topically.

The rheological and structural characteristics of carbomer-based gels are significantly influenced by their pH, formulation components, and interactions within the matrix. The literature data [42,43] revealed that, as pH increases, carbomer-based gels demonstrate enhanced structural viscosity and adhesivity. The literature studies demonstrate that the release of carbomer gels is higher at alkaline pH values [42], highlighting the importance of pH in determining the physical and biopharmaceutical properties of the gel [43].

#### 2.2.2. Spreadability

**Figure 9:**
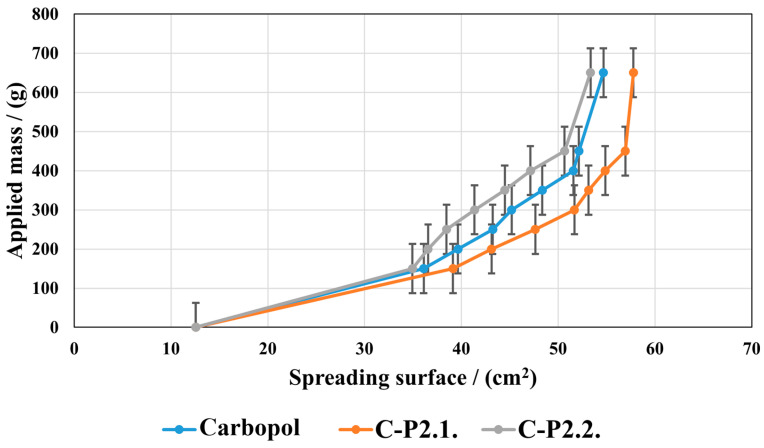
The hydrogels’ spreading properties.

Figure 9 presents how the spreading surface changes as the applied mass increases.

P2.1 induces an increase in the spreading ability compared to the Carbopol base gel, and P2.2 a decrease. However, the two porphyrins do not lead to significant changes in the gel structure; the values of the spreading areas within the three series are close to each other. In all the gels tested, the spreading area increases with the increase in the mass applied, indicating a good spreading performance on the skin, resulting in easy and uniform application.

Enhanced spreadability, as demonstrated by our hydrogels, improves the ease of application and skin coverage, leading to greater acceptance by patients. In addition, formulations that are easier to spread can cover more skin, a fact that could improve their therapeutic effect [44]. The analysis of our developed gels’ extensibility shows the strength of the formulation (firmness), the tackiness, and the adhesion. The maximum positive force that can deform a gel is its firmness, and this can be used to determine the strength of the formulation [45,46,47]. It can be assumed that a large number of physicochemical factors combine to produce bioadhesion of carbomer gels [48]. These factors include hydrogen bonding, van der Waals forces, hydrophobic interactions, and other attractive forces such as the physical entanglement of polymer chains by diffusion processes and electrostatic interactions that can be repulsive or attractive. It seems that enhanced cohesiveness may also be significantly influenced by the gels’ intrinsic rheological qualities, like the adhesive performance, which is highly dependent on the matrix structure and directly influences the swelling behavior [49]. For developed gels, we can assume that the adhesive capacity is critical for topical delivery to guarantee retention on the skin surface, as demonstrated in the literature [50].

#### 2.2.3. In Vitro Adhesion Ability

There are no significant differences between the adhesive properties of the Carbopol base gel and the porphyrin-containing hydrogels. For the base gel, the required force is 30 g/cm^2^, similar to that of C-P2.1 (29 g/cm^2^) and C-P2.2 (32 g/cm^2^). PEG 200 could be responsible for the small differences between the hydrogels, as it is known to be a major plasticizer; however, considering that it is used in the same amount in both porphyrin gels and that there is a negligible decrease for both C-P2.1 and C-P2.2, it is clear that the type of porphyrin minor influences the cohesiveness of the gelled systems.

#### 2.2.4. Rheology Measurements

**Figure 10:**
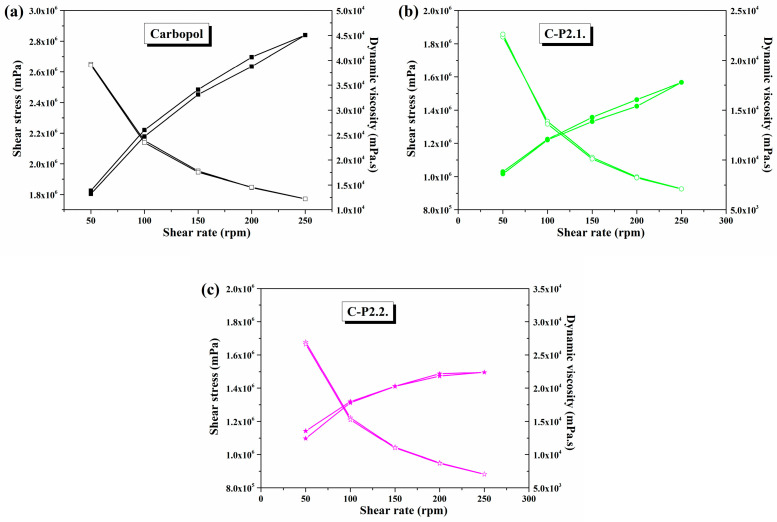
Variation in dynamic viscosity and shear stress as a function of shear rates in (**a**) Carbopol, (**b**) C-P2.1, and (**c**) C-P2.2.

Figure 10a–c represent the data obtained from the rheology analysis.

The three hydrogels show a pseudoplastic behavior characteristic of gelled systems, in which the dynamic viscosity decreases and the shear stress increases with increasing shear rate (Figure 10). With comparable shear stress and dynamic viscosity in both the upward and downward direction of the shear rate, all samples exhibit thixotropic properties.

The incorporation of porphyrins into the gel matrix leads to a significant reduction in the viscosity of the Carbopol gel. P2.1 (Figure 10b) causes a stronger decrease in viscosity compared to P2.2 (Figure 10c), a phenomenon that confirms a change in the system structure and, thus, also in the viscoelastic behavior. Nevertheless, both porphyrins change the internal structure of the material and lead to new types of interactions and a restructuring of the spatial distribution [51,52,53].

In comparison to the abovementioned studies, all three developed hydrogels exhibit thixotropic behavior with typical viscoelastic properties for carbomer dispersions [54]. The disruption of the system microstructure is obvious when porphyrins are added, a phenomenon that is due to the formation of new interactions between the substrates that are probably hydrophilic in nature [55]. Nevertheless, the influence of PEG 200 on the flow behavior cannot be overlooked. Several studies have shown that adding polyethylene glycol or other solvents to a carbomer matrix gel influences the properties of the hydrogen bonds among the polymer, solvent, and water, thus affecting the viscoelastic characteristics of the gel [56,57,58]. Since the dynamic viscosity decreases after the addition of porphyrins, and PEG 200 solutions are included, it can be concluded that the attraction forces between dispersed particles are reduced. Deformation-related modifications in the gel morphology, the orientation of the polymer chain segments, and the reduction in the number of entanglements between polymer chain segments and side chains can all alter the gel network structure [56]. For C-P2.1 and C-P2.2, a structural dynamic decrease in the free energy of the hydrogels is achieved. This is shown by the decrease in viscosity, causing them to become more fluid compared to the Carbopol gel base [59].

The flow behavior and viscoelastic properties of gels are of great importance, as they allow for a general prediction of the stability, spreadability, and deformability of topical gel systems. For the present study, it was found that the rheological properties of the gel were affected by the addition of porphyrins [60]. Even if not a significant change, it led to a decrease in the adhesive properties of the hydrogels. The hysteresis curves and flow behavior of carbomer-based gels are essentially consistent with the results of other studies [61]. The recommended thickness range for bioadhesive films is 0.05 to 1.0 mm [62]. The polymer choice has a significant impact on the formation of the film, as the polymer chains interact with each other during solvent evaporation to form a solid polymer matrix that facilitates drug release and uniform adhesion to the skin’s surface [63,64]. Since the hydrogel’s viscosity increases as the solvent evaporates, it also has a significant impact on the thickness of the film [65]. Mechanical strength and elasticity are highly influenced and are also impacted by the active ingredient’s physicochemical properties and the type of dispersion. The firm films might break apart on the skin surface, and this can cause discomfort, irritation, and even drug loss [66]. In this case, the carbomer films’ appropriate elasticity is supported by the tensile strength. Interpenetrating polymer chains significantly increase the elongation at break. The amorphous nature and high water content further support the elastic and flexible film structure. The addition of a plasticizer increases the elongation of the film due to the amorphous composition of the gel [67,68]. The role of the plasticizer is to change the viscoelastic properties of the polymer, in turn affecting the adhesion and release behavior of the material.

#### 2.2.5. Dry Gel Evaluation

Carbopol-based films have a thickness of 0.10 ± 0.002 mm, with no difference between gel types. Since the thickness of the film is directly related to the concentration of the drug and the polymer’s ability to bind biologically, uniformity is essential [62,69]. The values for mechanical strength are 0.38 kg/mm^2^ for C-P2.1, 0.36 kg/mm^2^ for C-P2.2, and 0.41 kg/mm^2^ for the Carbopol base gel.

In contrast, the elongation of the Carbopol gel decreases proportionally to the mechanical strength after porphyrins are introduced into the matrix, from 8% for the 1% Carbopol gel to 6% for C-P2.1 and 5% for C-P2.2.

The introduction of porphyrins into the Carbopol gel structure leads to a considerable increase in moisture content, explaining the low viscosity of these systems. While dry Carbopol gel contains 6.33% moisture, C-P2.1 contains 8.14% and C-P2.2 contains 8.86%. The addition of P2.2. to the hydrogel matrix increases the amount of retained water, a phenomenon that is probably due to the change in the gel’s structure [70,71,72,73,74].

The measurement of tensile strength represents the resistance of the film, determined by the type and quantity of plasticizers and polymers used. When the chain flexibility in water-soluble polymers decreases, the cross-linking density increases and prevents overhydration [75]. The mechanism of bioadhesion is improved by functional groups with hydrogen bonding capabilities. The literature demonstrates that a soft and weak polymer has low tensile strength and elongation values, a hard and brittle polymer usually has medium tensile strength and low elongation values, and a soft and tough polymer has high tensile strength and elongation values [76]. Considering these aspects, our studies establish that polymers with balanced tensile strength and elongation values are optimal for topical applications. For topical hydrogels, the mechanical characteristics of the films are of crucial importance, as they show how resilient the substance is to changes in shape without breaking. Plasticizers can penetrate between the polymer chains, thus interacting with the functional groups of the polymer. The bonds between the polymer chains become weaker as the intermolecular pressures between them decrease, in turn increasing the flexibility of the film [77,78].

**Figure 11:**
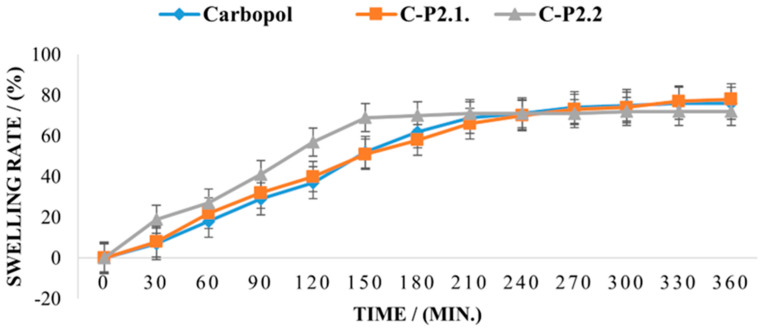
The swelling rate of the studied samples.

Figure 11 displays the rate of swelling over 6 h.

The swelling capacity for the 1% Carbopol gel exceeds 80% after 360 min, and the effect of P2.1 on the swelling behavior of the matrix is insignificant, indicating that the moisture absorption performance is polymer-controlled and no new interparticle bonds are formed in C-P2.1. In contrast, the swelling behavior of C-P2.2. is different, with a higher swelling capacity in the first 150 min; then, a plateau is reached, and no further moisture can be absorbed. The swelling behavior of a dry hydrogel is mostly dependent on its composition and structure and also affects its mucoadhesive properties. When the hydration degree increases, mucoadhesion becomes more cohesive, up to a point, then loosening at the polymer–tissue interface and leading to excessive hydration, which causes an abrupt drop in mucoadhesion [49,50]. It has been demonstrated that the addition of compounds with a high proportion of carboxylic acid or hydroxy groups in the carbomer matrix is the cause of the rise in moisture content [79] and that, conversely, the compactness of the film network can be the cause of the decrease in moisture content when this occurs [80].

The stability, resistance to microbiological growth, and unbreakability of the hydrogel film depend on the adequate moisture content of the formulations [81,82]. Also, the degree of particle swelling is determined by the balance between the internal elasticity of the gel particles and the osmotic pressure of the free counterions [83]. The modeling of equilibrium swelling of anionic hydrogels is considered an elastic three-phase media with a solid polymer, an aqueous solvent, and mobile ions as solutes [84,85]. Several studies have shown that the swelling ratio of carbopol molecules decreases significantly in PEG 400 solutions [86,87]. Because the PEG 200 solution used to solubilize the two porphyrins and added to the gel network represents a small amount, its influence on the swelling capacity appears to be minor in the present study. Also, the same authors observed that the use of PEG 400 has a low influence on the interactions between the particles in the limit of small deformations, but it obviously impacts the swelling of the Carbopol molecules.

The osmotic pressure between the external solvent and the internally charged environment controls the final degree of swelling in a cross-linked structure, such as the Carbopol molecules, even though electrostatic repulsions may contribute to an initial disentanglement of the collapsed structure. These results demonstrate the importance of the kinetics of the swelling process in achieving the desired swollen state. The swelling kinetics of these systems are diffusion-controlled. This means that smaller molecules, which have a stronger affinity for the polymer and a higher capacity to transfer charges, move preferentially within the cross-linked structure of Carbopol [88]. Also, chemical and physical factors such as pH, temperature, ionic strength, solvent, and external electrical force can influence the characteristics of a Carbopol gel. The physical and rheological properties of carbomer polyelectrolyte gels are primarily determined by their chemical structure and properties [89]. The results of the molecular dynamics simulations for internal osmotic pressure and gel swelling enable the determination of thermodynamic quantities such as the equilibrium swelling ratio and the osmotic pressure [90]. This explains the influence of P2.1 and P2.2 PEG 200 solutions, respectively, on the carbomer gel network.

The low aqueous solubility, the tendency to aggregate in biological environments, and the limited tumor selectivity of porphyrins cause limitations for dermato-oncological applications. Aggregation is particularly problematic, as it can quench fluorescence and reduce the generation of singlet oxygen, the primary cytotoxic agent in PDT. Structural modifications, such as the addition of polar functional groups, can improve solubility, reduce aggregation, and enhance photodynamic activity. For example, the porphyrin derivatives P2.1 and P2.2 used in this study have been chemically optimized to improve these characteristics. P2.2, in particular, has shown favorable solubility, selective tumor accumulation, strong singlet oxygen production, and minimal cytotoxicity, making it a promising candidate for PDT. Compared to our previous research, in which the same P2.1 and P2.2 were incorporated into a hydroxypropyl cellulose (HPC) hydrogel, some aspects can be mentioned: (i) Carbopol, a synthetic cross-linked polymer, is known for its ability to form gels with high viscosity and bioadhesive properties; (ii) hydroxypropyl cellulose, a natural polymer, has good solubility in water and the ability to form clear, non-irritating gels; (iii) while previous research with HPC-based systems has provided valuable information on porphyrin delivery, Carbopol’s unique properties, such as its superior bioadhesion and rheological characteristics, offer new opportunities for improving the localized delivery and sustained release of photosensitizers in dermatological applications.

## 3. Materials and Methods

### 3.1. Materials

The used porphyrins (P2.1 and P2.2) were synthesized following methodologies previously established and detailed [20]. Carbomer 940 and triethanolamine (TEA) were purchased from Fagron, Trikala, Greece. A Mettler Toledo AT261 balance (with a sensitivity of 0.01 mg, Mettler Toledo, Greifensee, Switzerland) was used to weigh the ingredients.

### 3.2. The Development of Hydrogels

Carbomer 940 is an anionic synthetic polymer with a high molecular weight and high water solubility that forms stable hydrogels at pH values above 5.5. The gels formed have high mucoadhesive properties [86], are safe and effective for topical application, are non-irritating, and do not interact with other ingredients [91].

The 1% carbomer hydrogel was prepared by dissolving the carbomer in water and stirring at 900 rpm at room temperature using a Heidolph MR 3001K magnetic stirrer (Schwabach, Germany). The resulting dispersion was stored in a refrigerator at 5 °C for 24 h. Triethanolamine was then gradually added while stirring continuously at 900 rpm until a translucent gel was obtained. The gel produced served as the base for the porphyrin gels and as a reference for the subsequent investigations.

The porphyrin hydrogels were prepared by incorporating 1 mL of each 10 mM porphyrin solution in PEG 200 into 50 g of carbomer gel base by stirring in the dark with a magnetic stirrer at 750 rpm at room temperature. Finally, two hydrogels (C-P2.1 and C-P2.2) containing 10 µg porphyrin/g were prepared.

The obtained gels’ characteristics were assessed in relation to the 1% Carbopol hydrogel.

### 3.3. Physico-Chemical Evaluation

A JASCO FT/IR 4700 spectrophotometer (Tokyo, Japan) was used to obtain Fourier-transform infrared (FTIR) spectra in the wavenumber range of 4000 to 400 cm^−1^. The spectrophotometer was equipped with a monolithic diamond-attenuated total reflectance (ATR) accessory (Tokyo, Japan). Measurements were taken at a 45° incident angle, with 64 accumulations and a resolution of 4 cm^−1^. A Rigaku Ultima IV diffractometer (Rigaku Co., Tokyo, Japan), equipped with parallel beam geometry and CuKα radiation (λ = 1.5406 Å), was employed to obtain X-ray diffraction (XRD) diffractograms. Data were collected within a 2θ range of 5° to 60°, using a scanning speed of 2°/min with a step size of 0.02°. Thermogravimetric analysis (TGA) was accomplished using a Mettler Toledo TGA/SDTA851^e^ thermogravimeter (from Mettler-Toledo, Greifensee, Switzerland). The thermal experiments were carried out between 25 °C and 600 °C. Measurements were conducted under a synthetic air flow of 80 mL/min. The used heating rate was 10 °C/min.

Atomic force microscopy (AFM) was performed in non-contact mode using an XE-100 from Park Systems (Park Systems Corporate, Suwon, Republic of Korea), which features decoupled sample/probe scanners. NSC36B tips (MikroMasch, Sofia, Bulgaria) were used in all AFM measurements, with a curvature radius below 8 nm, a cone angle of approximately 40°, a tip height of ~15 µm, a force constant of ~2 N/m, and a resonance frequency of 130 kHz. AFM samples were prepared by placing a droplet of the hydrogels on clean microscope glass slides (Heinz Herrenz, Hamburg, Germany) and allowing them to dry at room temperature. Images were processed using the XEI software (v 1.8.0, Park Systems) and the Scanning Probe Image Processor software SPIP™ v. 4.6.0.0 (Lyngby, Denmark) [92] for roughness (Rq and Rpv) as well as mean fractal dimension (MFD) and texture direction index (Stdi) evaluation. The root mean square roughness (Rq) represents the standard deviation of the height value, while the peak-to-valley parameter (Rpv) is the height difference between the lowest and highest points in the scanned area.

UV-vis spectroscopy measurements were performed using a LAMBDA 35 UV/vis spectrophotometer (Perkin Elmer Life and Analytical Science^®^, Waltham, MA, USA). The instrument, equipped with both deuterium and tungsten lamps for light source switching, offers high precision, with a wavelength accuracy of ±0.1 nm at the D2 peak (656.1 nm) and reproducibility within ±0.05 nm for 10 measurements. The optics system employs a single-beam, lens-free design with quartz-coated mirrors to minimize chromatic aberrations. Samples were analyzed in 1 cm quartz cuvettes without dilution, and thermal stability was ensured using a FALC^®^ circulating water bath set to maintain a constant temperature of 37 °C [39,93].

Fluorescence spectroscopy was conducted using a JASCO^®^ FP-6500 spectrofluorometer (JASCO Co., Ltd., Kyoto, Japan), featuring a 1 nm resolution and a signal-to-noise ratio of 200 or higher. The instrument has a shielded 150 W Xenon lamp as the light source. Raman band sensitivity (3200:1 at 350 nm excitation) and 1 nm resolution were maintained for both excitation and emission. Excitation was performed at 426 nm, as determined by the UV-vis spectra, and thermal regulation during measurements was maintained at 37 °C using an Advantage–Lab AL03-10^®^ thermal circulator [40].

### 3.4. Pharmacotechnical Assessment

#### 3.4.1. pH Measurement

A total of 1 mL of distilled water (pH 6.5 ± 0.5) was mixed with 0.2 g of each hydrogel, and the pH values were measured using the electrode of a CONSORT P601 pH meter (produced by CONSORT^nv^, Turnhout, Belgium).

#### 3.4.2. Spreadability

On a glass plate, 1 g of each hydrogel was placed in the center of a circle with a diameter of 2 cm. After a second glass plate with a 150 g weight was placed on the surface, the circle’s diameter filled with the gel was measured. After resting for 2 min, weights of 50 g, 100 g, 150 g, 200 g, 250 g, 300 g, and 500 g were successively placed on the upper glass plate. The diameter of the circle occupied by the gel was then calculated. The following formula was used to determine the spreading area occupied by the hydrogel:

(1) $$S=\pi r^{2}$$

#### 3.4.3. In Vitro Adhesion Ability

A thin layer of hydrogel was spread on a clamp of a digital tensiometer (LR 10K Plus, West Sussex, UK) with a size of 1 cm^3^. The second clamp was then used to compress the hydrogel. The mass needed to remove the second clamp from the gel surface was measured, while the test was conducted at 100 mm per minute. The adhesion ability was measured by calculating the tensile force required to remove the second clamp from the entire hydrogel-covered surface. The equation used [94] is:

(2) tensile force gmm2=detaching mass gsurface mm2

#### 3.4.4. Rheology Measurements

The analysis was carried out on 50 g of each hydrogel using a B-one Plus rotational viscometer from Lamy Rheology Instruments, Champagne du Mont d’Or, France, equipped with an RV7 measuring spindle. At 22 °C, the rotation speeds were increased from 50 rpm to 250 rpm and then repeated in a decreasing order. There was no pause between two consecutive determinations; the time for each determination was set at 150 s. The hysteresis curve for shear stress and dynamic viscosity was plotted.

#### 3.4.5. Dry Gel Evaluation

To ascertain the gels’ more objective performance subsequent to skin application, the hydrogels were thinly spread in Petri dishes and allowed to dry for 48 h at room temperature (22 °C). After peeling off the dried films, their mechanical and swelling characteristics were investigated.

#### 3.4.6. Mechanical Properties

##### Thickness

The thickness of the produced film was measured using a digital micrometer from Yato Trading Co., Ltd., Shanghai, China, with a measurement range of 0 to 25 mm and a resolution of 0.001 mm.

##### Tensile Strength and Elongation

A digital tensile strength tester for universal materials from Lloyd Instruments Ltd., LR 10K Plus, West Sussex, UK, was used to evaluate tensile strength and elongation behavior. The test was performed at a speed of 3 mm/second at a distance of 20 mm. The breaking force could be determined by aligning the films vertically between the two braces [75]. The elongation at break and tensile strength were calculated using the following formulas:

(3) tensile strength kgmm2=force at breakage kgfilm thickness mm×film width mm

(4) $$elongation\;\left(\%\right)=\frac{increase\;in\;film\;length}{initial\;film\;length}\times 100$$

##### Moisture Content

The thermogravimetric approach was employed to evaluate the drying loss using a Mettler-Toledo HR 73 halogen humidity analyzer (Mettler-Toledo GmbH, Greifensee, Switzerland) [95].

##### Swelling Ratio

The swelling ratio was determined by weighing 0.2 g of the film, formed by the dried hydrogel, every thirty minutes during a six-hour incubation period at 37 ± 1 °C on Petri dishes containing 1.5% agar gel.

The swelling ratio was calculated using the following formula:

(5) $$swelling\;ratio=\frac{w_{t}-w_{i}}{w_{i}}\times 100$$

where *w_t_* is the patch weight at time *t* after the incubation and *w_i_* is the initial weight [96,97,98].

##### Statistical Analysis

The spreadability and swelling rate are stated as the mean ± SD (standard error of the mean of *n* = 3). In the figures, error bars are included, calculated as standard errors or standard deviations, suitable for each dataset.

## 4. Conclusions

The present research successfully establishes the effective integration of the two developed porphyrin structures, i.e., 5,10,15,20-tetrakis-(4-acetoxy-3-methoxyphenyl) porphyrin, noted as P2.1, and 5-(4-hydroxy-3-methoxyphenyl)-10,15,20-tris-(4-acetoxy-3-methoxyphenyl), noted as P2.2, respectively, into a 1% Carbopol hydrogel matrix. Complete physicochemical and pharmacotechnical assessments were used to evaluate the appropriateness of the developed porphyrin-doped hydrogels for biomedical applications. The following conclusions can be made:

(i) The physicochemical characterization using FTIR, XRD, TGA, AFM, UV-vis, and fluorescence analyses demonstrates the complete incorporation of the two porphyrins in the 1% Carbopol hydrogel matrix.
(ii) The incorporation of P2.1 had a more visible effect on the rheological behavior of the Carbopol gel compared to the incorporation of P2.2, leading to a decrease in the viscosity of the Carbopol gel. These findings highlight the impacts of the two porphyrin structures on the gel’s internal structure.
(iii) Mechanical characterization revealed that both hydrogel formulations exhibited desirable mechanical properties, indispensable for ensuring their stability and adherence to the skin surface during application.
(iv) Additionally, the hydrogels maintained a pH range suitable for the required topical applications, enhancing skin safety by ensuring good tolerability.
(v) The hydrogels displayed high swelling capacities, indicative of their ability to absorb moisture effectively in the first 150 min. This property is advantageous for maintaining a hydrated environment on the skin, a phenomenon that is particularly beneficial for wound care and for enhancing drug penetration.

In conclusion, both P2.1 and P2.2 are suitable for incorporation into the Carbopol gel matrix due to their rather similar superior solubility, selective accumulation in tumor cells, and favorable rheological and photodynamic properties.

These characteristics are critical for maximizing the therapeutic efficacy of the hydrogels in PDT by facilitating optimal light penetration and interaction with the porphyrin compounds. In conclusion, the results of this study highlight the potential use of porphyrin-doped 1% Carbopol hydrogels as innovative carriers, particularly for topical photodynamic therapy applications to treat malignant skin cancers and other conditions, including inflammatory disorders and cutaneous infections. By conserving or enhancing the main physicochemical and mechanical properties, these formulations address significant challenges associated with the transport of porphyrin derivatives, including stability and bioavailability.

## Figures and Tables

**Table 1:** Thermal data obtained from TGA curves.

Compound	1st Step (Temperature and Mass Loss)	2nd Step (Temperature and Mass Loss)	Remaining Mass at 600 °C
Carbopol	Below 150 °C/11.6%	*T*_DTA_ = 206.3 °C and *T*_DTG_ = 205.3 °C *T*_DTA_ = 375 °C and *T*_DTG_ = 374.6 °C *T*_DTA_ = 528.1 °C	3%
C-P2.1	Below 150 °C/8.6%	*T*_DTA_ = 228 °C and *T*_DTG_ = 253 °C *T*_DTG_ = 447 °C and *T*_DTG_ = 443 °C	3.26%
C-P2.1	Below 150 °C/10%	*T*_DTA_ = 240 °C and *T*_DTG_ = 233.1 °C *T*_DTG_ = 508 °C and *T*_DTA_ = 509.6 °C	2.9%

**Table 2:** Spectral characteristics of porphyrins P2.1 and P2.2 in 1% Carbopol gel.

Gel	Absorption λ_max_ (nm)					Emission λ_max_ (nm)
	Soret Band	Q_y_ (1.0)	Q_y_ (0.0)	Q_x_ (1.0)	Q_x_ (0.0)	
C-P2.1	427	516	550	595	645	652
C-P2.2	424	519	555	590	650	654

## Data Availability

Data are contained within the article.
